# Let-7e-5p Regulates IGF2BP2, and Induces Muscle Atrophy

**DOI:** 10.3389/fendo.2021.791363

**Published:** 2021-12-24

**Authors:** Takuro Okamura, Hiroshi Okada, Yoshitaka Hashimoto, Saori Majima, Takafumi Senmaru, Naoko Nakanishi, Mai Asano, Masahiro Yamazaki, Masahide Hamaguchi, Michiaki Fukui

**Affiliations:** ^1^ Department of Endocrinology and Metabolism, Graduate School of Medical Science, Kyoto Prefectural University of Medicine, Kyoto, Japan; ^2^ Department of Diabetes and Endocrinology, Matsushita Memorial Hospital, Moriguchi, Japan

**Keywords:** micro RNA, let-7e-5p, muscle atrophy, Igf2bp2, sarcopenia

## Abstract

**Background and Aims:**

To understand the role of microRNAs in muscle atrophy caused by androgen-depletion, we performed microarray analysis of microRNA expression in the skeletal muscles of Sham, orchiectomized (ORX), and androgen-treated ORX mice.

**Methods:**

To clarify role and mechanisms of let-7e-5p in the muscle, the effect of let-7e-5p overexpression or knockdown on the expression of myosin heavy chain, glucose uptake, and mitochondrial function was investigated in C2C12 myotube cells. Moreover, we examined serum let-7e-5p levels among male subjects with type 2 diabetes.

**Results:**

We found that the expression of the miRNA, lethal *(let)-7e-5p* was significantly lower in ORX mice than that in Sham mice (p = 0.027); however, *let-7e-5p* expression in androgen-treated ORX mice was higher (p = 0.047). Suppression of let-7e-5p significantly upregulated the expression of myosin heavy chain, glucose uptake, and mitochondrial function. Real-time PCR revealed a possible regulation involving let-7e-5p and *Igf2bp2* mRNA and protein in C2C12 cells. The serum let-7e-5p levels were significantly lower, which might be in compensation, in subjects with decreased muscle mass compared to subjects without decreased muscle mass. Let-7e-5p downregulates the expression of *Igf2bp2* in myotube cells and inhibits the growth of the myosin heavy chain.

**Conclusions:**

Based on our study, serum level of let-7e-5p may be used as a potential diagnostic marker for muscle atrophy.

## Introduction

Sarcopenia, an aging-related condition characterized by the loss of muscle mass, strength, and function, is an important global health concern (1). Sarcopenia-associated muscular atrophy not only impairs motor function, but also increases the likelihood of falls and fractures, affects daily activities (2, 3), and increases the risk of mortality (4). The pathogenesis of skeletal muscle atrophy includes reduced regenerative capacity of muscle satellite cells, reduced protein synthesis, and accelerated degradation of myotube cells (5). Although the exact mechanism is unknown, reduced androgen production has been implicated in the pathogenesis of skeletal muscle atrophy. Roy et al. reported an association between serum androgen concentration and skeletal muscle mass and strength (6). In addition, Basualto-Alarcón et al. showed that androgen signals induce muscle hypertrophy through the mTOR and androgen receptor pathways (7). However, androgen replacement therapy has been reported to have several adverse effects (8). Therefore, there is an urgent need to develop new therapeutic options to prevent androgen deficiency-induced skeletal muscle atrophy.

Micro RNAs (miRNAs) are short single-stranded non-coding RNAs approximately 19–23 nucleotides in length that regulate gene expression in the post-transcriptional control phase of target messenger RNAs (mRNAs). MiRNA-mediated transcriptional repression is known to play an important role in biological processes such as cell proliferation and differentiation (9), apoptosis (10), metabolism (11), and development (12). MiRNAs bind to their target mRNAs with incomplete homology, thereby destabilizing the target mRNA, and inhibiting protein synthesis (13).

Recent studies have shown that a single miRNA can regulate the expression of multiple genes associated with a single pathology. Abnormal expression of miRNAs in the skeletal and cardiac muscles is associated with muscle damage (14). We previously reported that activation of the Akt-mTOR pathway, caused by miR-23b-3p overexpression-mediated PTEN repression, counteracts skeletal muscle atrophy and has beneficial effects on the skeletal muscles, including increased expression of myosin heavy chain, myoD, and myogenin, and increased glucose uptake and ATP activity (15). In contrast, in this study, using microarray analyses of miRNA in skeletal muscles, we found that the miRNA, lethal (let)-7e-5p, was overexpressed in androgen-treated orchidectomized (ORX) mice, compared to ORX mice. Let-7 family has been reported to be related with muscle atrophy. Muscle biopsy studies found that the expression of let-7b and let-7e was increased in the skeletal muscle in older men with less lean mass compared to young men (16). In another study, in healthy men with 21-days bed rest let-7 in muscle was increased (17). On the other hand, the expression of let-7 was decreased in skeletal muscle at 10-days of bed rest in healthy man (18), and the expression of the let-7 family within atrophied skeletal muscle has been reported in various ways and no clear conclusions have been reached. In this study, we investigated the association between skeletal muscle atrophy and let-7e-5p using murine C2C12 myotube cells. In addition, we examined the relationship between let-7e-5p levels in human serum and muscle atrophy.

## Materials and Methods

### Animals and Experimental Design

All experimental procedures were approved by the Committee for Animal Research, Kyoto Prefectural University of Medicine (M2020-40). Six-week-old C57BL/6 J male mice were purchased from Shimizu Laboratory Supplies (Kyoto, Japan) and housed in specific pathogen-free controlled environment. The mice were fed a standard diet (SD; 344.9 kcal/100 g, fat kcal 4.6%; CLEA Japan, Tokyo) for 4 weeks starting at 8 weeks of age.

Mice were either orchiectomized (ORX group) or sham-operated (Sham group) at 7 weeks of age. Under isoflurane inhalation anesthesia, the skin of the testicle area is cut by 1 cm, the testicle is removed, and compression hemostasis is applied until obvious bleeding stops. In the sham group, the skin at the testicle area is cut by 1 cm, and the testicle is not removed, and only the skin is sutured. For the androgen-treatment group (ORX+A), 8-week-old ORX mice were treated with testosterone, administered once every 2 days for 4 weeks *via* subcutaneous injection of androgen enanthate (3.6 μg/g body weight; ISEI, Yamagata, Japan) diluted in sesame oil (19).

When the mice reached 12 weeks of age, mice were sacrificed by the administration of a combination of anesthetics including 0.3 mg/kg medetomidine, 4.0 mg/kg midazolam, and 5.0 mg/kg butorphanol (20). The soleus muscle was obtained, frozen immediately, and stored at -80°C until use. QIAzol Lysis reagent (Qiagen, Hilden, Germany) was used for miRNA extraction.

### MiRNA Microarray Analysis

The soleus muscles were obtained from the mice in the ORX, ORX+A, and Sham groups, and subjected to GeneChip miRNA 4.0 Array (cat. #902412, Applied Biosystems, Foster City, CA, USA). The relative abundance of the miRNAs within the groups was evaluated using the weighted average distance (WAD) method using R (21) and paired t-tests. The WAD method ranked the genes based on high expression, high weightage, or fold-change. WAD was found to be an effective method of transcriptome analysis. The data were preprocessed with Robust Multichip Average normalization, and the global miRNA expression was visualized as a volcano plot.

### Mouse Skeletal Muscle Cell Culture

C2C12 cells (a mouse myoblast cell line; KAC Co. Ltd., Kyoto, Japan), were plated in 24-well plates and grown in Dulbecco’s modified Eagle’s medium (DMEM) supplemented with 20% fetal bovine serum (day 2). The medium was changed every other day. When the cells reached 80% confluence, they were differentiated in DMEM supplemented with 2% horse serum (differentiation medium) (day 0). At 24 h after the medium change, the cells were transfected with 30 nM let-7e-5p mimic/inhibitor or a scrambled sequence (mirVana ^®^), which were purchased from Thermo Fisher Scientific, using X-treme Gene siRNA transfection kit (Roche, Mannheim, Germany) according to the manufacturer’s recommendations (day 1). At 96 h (day 5) post-transfection, the myotube cells were evaluated through various experiments.

### Gene Expression in C2C12 Cells

Gene expression in the C2C12 cells was analyzed on day 5. Medium was removed and the cells were washed with cold phosphate buffered saline (PBS) twice. Cells were detached from the dish using cell scrapers, homogenized in ice-cold QIAzol Lysis reagent, and total RNA was isolated following the manufacturer’s instructions. Total RNA (0.5 μg) was reverse transcribed into cDNA using a High-Capacity cDNA Reverse Transcription Kit (Applied Biosystems) with oligodT and random hexamer primers according to the manufacturer’s recommendations. The reverse transcription reaction was performed for 120 min at 37°C and the reaction was inactivated for 5 min at 85°C. We chose the *let-7e-5p* target mRNA, *Igf2bp2*, for further studies. We used real-time reverse transcription-polymerase chain reaction (RT-PCR) to quantify the mRNA expression of *Igf2bp2*, *Trim63*, *Fbxo32*, and *Hdac4*, which are involved in muscle atrophy (22). RT-PCR was performed using TaqMan Fast Advanced Master Mix (Applied Biosystems) according to the manufacturer’s instructions. The PCR conditions were as follows: 1 cycle of 2 min at 50°C and 20 s at 95°C, followed by 40 cycles of 1 s at 95°C, and 20 s at 60°C.

The relative expression of each target gene was normalized to the threshold cycle (CT) value of *Gapdh* quantified using the comparative threshold cycle 2−ΔΔCT method as described previously (23). Signals from the Sham mice were assigned a relative value of 1.0. Six mice were examined in each group, and RT-PCR was performed in triplicate for each sample. Total miRNA was extracted from the soleus muscle using the miRNeasy mini kit (Qiagen, Hilden, Germany).

For the miRNA RT-PCR experiments, cDNA was synthesized from 200 ng of total miRNA using a Taqman miRNA Reverse Transcription kit (Applied Biosystems). U6 was used as an endogenous control. RT-PCR was performed using TaqMan Fast Advanced Master Mix (Applied Biosystems) according to the manufacturer’s instructions. The PCR conditions used were as follows: 1 cycle of 2 min at 50°C and 20 s at 95°C, followed by 40 cycles of 1 s at 95°C, and 20 s at 60°C. We extracted and quantified let-7e-5p and U6 small nucleolar RNA using the 2−ΔΔCT method. Signals from the Sham mice were assigned a relative value of 1.0.

### Luciferase Reporter Assay

The putative let-7e-5p targets were predicted using TargetScan Human 6.2 (http://www.targetscan.org/). The putative recognition sites of let-7e-5p in the IGF2BP2 3’-untranslated region (3’-UTR) and its sequences are shown in **Supplementary Figure 2**. The reporter plasmids containing the wild-type (WT) 3′UTR (pmirGLO3-IGF2BP2-WT-3′UTR) and mutant (MUT) 3′UTR (pmirGLO3-IGF2BP2-MUT-3′UTR) were synthesised (Genecopoeia). C2C12 myotube cells in 96-well plates were transfected with 30 nM scrambled sequence (NC), a combination of let-7e-5p mimicking substrate (Applied Biosystems) and IGF2BP2 reporter vector or pEZX-MT01 control vector, which were purchased from Merk, using an X-treme GENE siRNA transfection kit according to the manufacturer’s instructions. All transfection experiments were performed in triplicate. Luciferase activity was assayed at 48 h after transfection, using a dual-luciferase reporter assay system (Genecopoeia).

### Protein Extracts and Western Blot Incubated With Antibodies

Whole C2C12 myotube cell extracts were prepared in a radio immunoprecipitation assay buffer (RIPA, ATTO, Tokyo; 50 mmol/L Tris (pH 8.0), 150 mmol/L NaCl, 0.5% deoxycholate, 0.1% SDS and 1.0% NP-40) containing a protease inhibitor cocktail (BioVision, Milpitas, CA, USA). Protein assays were performed using a BCA protein assay kit (Pierce/Thermo Scientific, Rockford, IL, USA) according with the manufacturer’s instructions. Total protein (20μg) was electrophoresed in 12% SDS-PAGE gels, and western blotting was carried out using standard protocols and proteins detected by ImageQuant LAS 500 (GE Healthcare, Piscataway, NJ, USA).

C2C12 myotube cells were subjected to protein extractions. First, 40–60 μg of protein extraction were incubated with the following primary antibodies; Igf2bp2 (1:1000), MY32 or gapdh (1:1500) diluted with EzBlock Chemi (ATTO, Osaka, Japan) overnight at 4°C, followed by incubation with goat anti-mouse IgG secondary antibodies conjugated to horseradish peroxidase diluted with EzBlock Chemi for 30 minutes at room temperature. All the antibodies listed in this section were obtained from Santa Cruz Biotechnology (Santa Cruz, Dallas, TX, USA).

### Immunocytochemistry

C2C12 cells were cultured in 8-well chamber slides and immunocytochemistry was performed on day 5. The cells were fixed in 4% paraformaldehyde and incubated with MY32, a primary monoclonal antibody against myosin heavy chain (Sigma-Aldrich, St. Louis, MO, USA), or F12B, anti-myogenin, (Sigma-Aldrich), diluted in PBS/1% BSA/0.3% TritonTM X-100 (Sigma-Aldrich) overnight at 4°C, and then with Texas-red-conjugated anti-mouse secondary antibody (Jackson ImmunoResearch) diluted in PBS/1% BSA/0.3% TritonTM X-100 overnight at 4°C for 1 h. The nuclei were stained with DAPI (Sigma-Aldrich). Images were captured with a BZ-X710 fluorescence microscope, and the fluorescence intensity of the cells and the nuclei count per image with a 20-fold magnification were analyzed using ImageJ (NIH). In addition, the fusion index was defined and determined according to a previous study (24).

### Apoptosis Detection by Flow Cytometry Analysis

C2C12 cells were cultured in 24-well chamber slides and flow cytometry analyses were performed on day 5 with Annexin V-FITC Apoptosis Detection Kit (Nacalai tesque, Kyoto, Japan) according to the manufacturer’s recommendations. FACS Canto II and FlowJo version 10 software were used for obtained data and analyzation.

### ATP Activity

C2C12 cells were cultured in 24-well plates, and cellular ATP was extracted on day 5 using an Intracellular ATP assay kit (Toyo-B-Net, Tokyo, Japan) according to the manufacturer’s instructions. The medium was removed, cells were washed twice with cold PBS, and then treated with ATP extraction buffer (400 μL/well) at room temperature for 5 min. The lysate was then dispensed into 96-well plates (on ice) in triplicate and luminescent reagent (100 μL/well) was added. ATP activity was quantitated as a measure of the luminescence using an Orion L microplate luminometer (Berthold Detection Systems, Pforzheim, Germany).

### 2-Deoxyglucose Uptake by C2C12 Cells

The uptake of [^3^H]2-deoxyglucose (PerkinElmer, Boston, MA) by C2C12 cells cultured in 24-well plates was measured. Cells were washed twice with serum-free DMEM, incubated in serum-free DMEM for 2 h at 37°C, and washed twice with PBS. Then, Krebs-Ringer-phosphate buffer (10 mM phosphate [pH 7.5], 113 mM NaCl, 5 mM KCl, 1.3 mM CaCl_2_, 1.2 mM MgSO_4_, and 1.2 mM KH_2_PO_4_ containing 0.3% BSA) was added in the presence or absence of 10 mU/mL bovine insulin for 30 min at 37°C. The uptake of 10 µM [^3^H]2-deoxyglucose was then measured over a 5-min period. Reactions were terminated by rapidly washing the cells twice with ice-cold Krebs ringer bicarbonate buffer.

Cells were then extracted using 0.2% SDS, and aliquots of the cell extract were counted by liquid scintillation and used to determine the protein concentration. Nonspecific uptake was measured in the presence of 10 µM of cytochalasin-B, and the values were subtracted from those corresponding to specific binding.

### Measurement of Cellular Oxygen Consumption Rate (OCR) in C2C12 Cells

OCR of C2C12 cells was determined using a Seahorse Extracellular Flux Analyzer XFp (Agilent Technologies, Santa Clara, CA). C2C12 cells (0.5 × 104 cells/well) were plated and cultured in normal medium in a Seahorse plate, and the experiment was conducted 5 days after changing the cells to differentiation medium. On the day of the measurement, the medium was replaced with XF assay medium supplemented with 10 mM glucose, 2 mM glutamine, and 1 mM pyruvate, and the cell culture plate was placed in a CO2-free incubator for 1 h. OCR was determined using a Seahorse Analyzer in combination with a Cell Mito Stress Test assay kit according to the manufacturer’s instructions. For the Cell Mito Stress Test, 2 μM oligomycin, 2 μM carbonyl cyanide 4-(trifluoromethoxy) phenylhydrazone (FCCP), and 1 μM rotenone /antimycin A were subsequently added to the assay medium to monitor different aspects of mitochondrial respiration. Two plates with the same conditions were prepared, one for the experiment and the other for cell counting after stripping the cells with trypsin EDTA, and OCR was normalized to the total number of cells.

### Study Population

The present study population was derived from the KAMOGAWA-DM cohort study, which is an ongoing prospective cohort study that began in 2014 (25). Approval for the study was obtained from the research ethics committees of the Kyoto Prefectural University of Medicine and Kameoka Municipal Hospital (E-466), and written informed consent was obtained from all the patients. For the present study, we collected information pertaining to male patients aged ≥ 65 years with type 2 diabetes, all of whom were Japanese, physically active, and KAMOGAWA-DM participants recruited from the outpatient clinic of the Kyoto Prefectural University of Medicine or Kameoka Municipal Hospital between August 2015 and September 2017. Patients with diabetic nephropathy stage 3 or higher (26) and those with inflammatory disease, malignancy, or endocrine disease were excluded from the study. Patients with class NYHA II–IV cardiac insufficiency (27) or severe chronic obstructive pulmonary disease were also excluded (27) as these conditions may influence the patient’s physical activity.

Next, we examined the decrease in muscle mass by determining each patient’s skeletal muscle index (SMI) based on the algorithms proposed by the Asian Working Group for Sarcopenia (28). The body composition of each patient was evaluated using a multifrequency impedance body composition analyzer (InBody 720, InBody Japan, Tokyo) (29), which is well correlated with the dual-energy X-ray absorptiometry (30). We obtained each patient’s body weight (BW, kg), body fat mass (kg), skeletal muscle mass (kg), and appendicular muscle mass (kg) and then calculated the skeletal muscle mass index (SMI) (kg/m2) by dividing the appendicular muscle mass (kg) by the square of the patient’s height (m) (30). The body mass index was defined as weight in kilograms divided by height in meters squared. The cut-off value for SMI was set to < 7.0 kg/m2. In this study, we selected a total of 32 male patients to form two groups of 16 patients with or without decreased muscle mass, matched for age. Approval for the study was obtained from the research ethics committees of the Kyoto Prefectural University of Medicine and Kameoka Municipal Hospital, and written informed consent was obtained from all the patients.

### Serum miRNA Extraction

RNA was extracted from serum samples using miRNeasy Serum/Plasma Kit (Qiagen) according to the manufacturer’s instructions. Briefly, 250 μL of serum was thawed on ice and centrifuged at 12,000 g at 4°C for 10 min to remove cellular debris. Thereafter, 200 μL of the supernatant was lysed in 1000 μL of QIAzol Lysis Reagent. After incubation for 5 min, 25 fmol of synthetic cel-miR-39 (Syn-cel-miR-39-3p miScript miRNA Mimic, Qiagen) was added to each sample as an external spiked-in control.

Total RNA, including small RNA, was extracted and eluted in 30 μL of RNase-free water using a QIAcube device (Qiagen). Serum levels of let-7e-5p and cel-miR-39 were examined by real-time PCR. A fixed volume of 2 μL of total RNA was reverse transcribed using Taqman miRNA Reverse Transcription kit (cat. #4366597, Applied Biosystems) in a total volume of 15 μL under the following cycling conditions: 16°C for 30 min, 42°C for 30 min, 85°C for 5 min, and then maintained at 4°C.

Real-time PCR was performed (in duplicate) using MiRNA Assay Kit and TaqMan Universal Master Mix II (no UNG; cat. #4440040, Applied Biosystems) on the StepOne Plus system (Applied Biosystems) under the following cycling conditions: 95°C for 10 min, followed by 40 cycles of 95°C for 15 s and 60°C for 1 min. The cycle threshold (Ct) values were calculated using StepOne Software v2.3 (Applied Biosystems). The miRNAs expression levels were normalized to those of cel-miR-39 determined using the 2^−ΔΔCt^ method.

### Statistical Analysis

Data were analyzed using JMP ver. 13.0 software (SAS, Cary, NC). Student’s t-test was used to compare the different groups. P = 0.05 was considered significant. Moreover, when necessary, Kruskal-Wallis tests followed by Bonferroni correction of the Mann-Whitney U test were used for multiple comparisons. In addition, the area under the curve (AUC) of serum let-7e-5p levels, for the presence of sarcopenia, was calculated by the receiver operating characteristic curve.

## Results

### MiRNA Array Analyses of Murine Soleus Muscle

**Figure 1:**
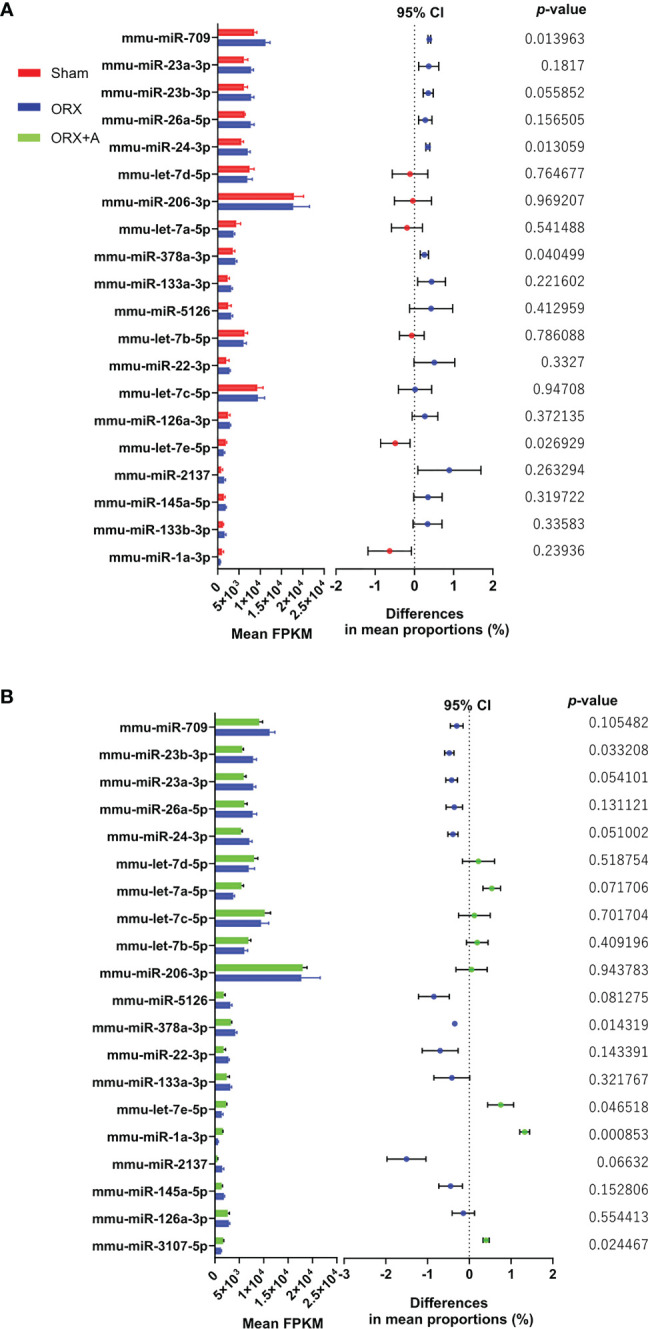
Top 20 miRNAs in the soleus muscle ranked using the weighted average difference method. Influence of miRNAs in Sham, ORX, and ORX+A mice was assessed using the weighted average differences method, and the assessed influence of the genera are ranked from top to bottom. Top 20 miRNAs are shown. Differences between these miRNAs were evaluated using paired t‐tests (n = 3). Left, histogram showing the mean fragments per kilobase of exon per million reads mapped (FPKM) of miRNAs (mean + standard deviation); right, 95% confidence interval (CI) of the differences in mean proportion and P‐ value by paired t‐test are shown. **(A)** Sham vs. ORX mice. **(B)** ORX vs. ORX+A mice. Sham: red, ORX: blue, ORX+A: green.

We examined the changes in miRNA expression in the soleus muscle of ORX mice by performing microarray analysis comparing the Sham and ORX mice administered with androgen (ORX+A). The differentially expressed genes in the Sham and ORX mice, and ORX and ORX+A mice were determined using the WAD algorithm, and the full ranking is shown in **Supplementary Tables 1** and **2**. Top 20 miRNAs were ranked from top to bottom, as shown in **Figures 1A, B**. Additionally, volcano plots and heatmap of microarrays data displaying the pattern of gene expression values for ORX mice versus sham or ORX+A mice were shown in **Supplementary Figures 1A, B** and **3**. In microarray analyses, the fold change of let-7e 5p expression in ORX mice was 0.73 ± 0.16 compared to that in sham mice, and 0.60 ± 0.12 (p =0.047). In real-time PCR, the expression of let-7e-5p was significantly reduced in the ORX mice, compared to that in Sham mice (p = 0.027), whereas replacement of testosterone restored the expression of let-7e-5p (p = 0.135) (**Supplementary Table 3**). In addition, the expression of let-7e-5p evaluated by RT-PCR in soleus muscle of ORX mice was significantly decreased, compared to that of sham or ORX+A mice (p =0.001), whereas that of ORX+A mice was restored to the same levels as that of sham mice (p =0.167) (**Supplementary Figure 1C**).

### Igf2bp2 Is a Potential Target of Let-7e-5p

**Figure 2:**
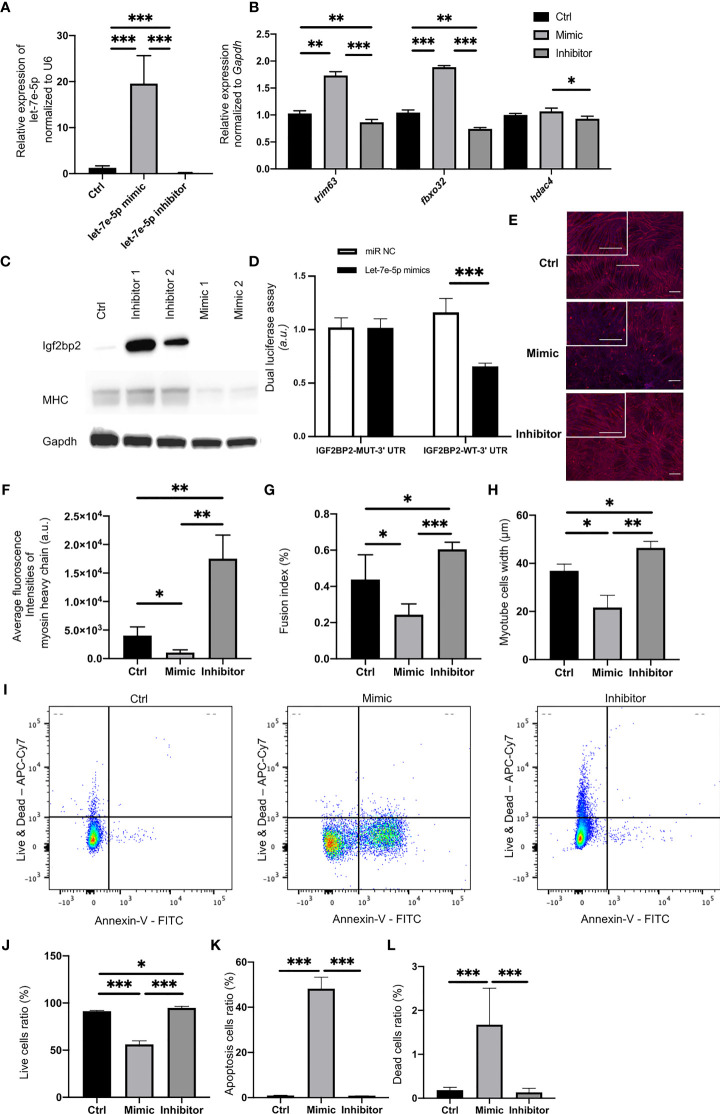
Let-7e-5p inhibits myotube formation in C2C12 cells *via* repression of Igf2bp2. **(A)** Let-7e-5p expression in C2C12 cells was assessed after transfection with a let-7e-5p mimic/inhibitor (30 nM) (n = 6). **(B)** Relative expression of mRNAs of indicated genes in C2C12 cells normalized to *Gapdh* expression (n = 6). **(C)** Western blotting to detect the levels of Igf2bp2 and MHC and Gapdh in C2C12 myotube cells transfected with a let-7e-5p mimic/inhibitor (n=6). **(D)** Target validation study by luciferase assay (n=6). **(E)** Immunocytochemistry in C2C12 cells (n = 6). *Red:* Myosin heavy chain. *Blue:* DAPI. Scale bar, 50 µm. **(F)** Fluorescence intensity of myosin heavy chain (arbitrary units, a.u.) was compared (n = 6). **(G)** Fusion indices of C2C12 cells are shown (n = 6). **(H)** Width of myosin heavy chain are shown (n=6). **(I)** Representative flow cytometry plots of C2C12 cells Annexin V- PI- live cells, Annexin V+ PI- apoptosis cells, and Annexin V+ PI+ dead cells. **(J)** The ratio of live cells is shown (n=6). **(K)** The ratio of apoptosis cells is shown (n=6). **(L)** The ratio of dead cells is shown (n=6). Data represent the means ± standard deviation (SD). *p = 0.05, **p = 0.01, ***p = 0.001 by one-way ANOVA. Kruskal-Wallis tests followed by Bonferroni correction of the Mann-Whitney U test were used for multiple comparisons. Ctrl: negative control, MHC: myosin heavy chain.

Putative let-7e-5p targets were predicted using TargetScan Human 6.2 (http://www.targetscan.org/). The putative let-7e-5p recognition sites in the 3’-untranslated region (3’-UTR) of insulin-like growth factor-2 mRNA-binding proteins 2 (Igf2bp2) and their sequences are shown in **Supplementary Figure 2**. We confirmed that the expression of let-7e-5p in C2C12 cells was significantly increased following transfection of the mimicking substrate, and decreased following transfection of an inhibitor (p = 0.001) (**Figure 2A**). Expression of *Igf2bp2* in C2C12 cells was significantly decreased following transfection with the let-7e-5p mimicking substrate (p = 0.001) (**Supplementary Figure 1D**). Conversely, the expression of Igf2bp2 in C2C12 cells was significantly increased by the let-7e-5p inhibitor (p = 0.001) (**Supplementary Figure 1D**). Moreover, in Western blot analyses, the Igf2bp2 protein levels were investigated. The Igf2bp2 protein levels of C2C12 cells transfected with the let-7e-5p inhibitor was higher than those with the let-7e-5p mimicking substrate (**Figure 2C**). In addition, the luciferase reporter assays using a 3’UTR of Igf2bp2 construct with let-7e-5p or a miR-Control construct expressing C2C12 myotube cells revealed a consistent reduction of luciferase activity in the let-7e-5p transfectants, suggesting that let-7e-5p represses Igf2bp2 directly (**Figure 2D**). The expression of genes related to muscle atrophy such as Trim63, Fbxo32, and Hdac4, was significantly increased in C2C12 cells transfected with the let-7e-5p mimicking substrate compared to that in cells transfected with the inhibitor (**Figure 2B**).

### Suppression of Let-7e-5p Upregulated the Expression of Myosin Heavy Chain in C2C12 Cells and Decreased the Apoptosis

In Western blot analyses, the MHC protein levels of C2C12 cells transfected with the let-7e-5p inhibitor was higher than those with the let-7e-5p mimicking substrate (**Figure 2C**). Next, we immunostained the cells with monoclonal antibodies against myosin heavy chain. Immunostaining revealed induction of growth in myotube cells transfected with the let-7e-5p inhibitor, while cells transfected with the let-7e-5p mimicking substrate atrophied (**Figure 2E**). In addition, the fluorescence intensity of myosin heavy chain in the C2C12 cells transfected with the let-7e-5p inhibitor was significantly higher than that in the cells transfected with the negative control (p = 0.006), whereas the level in cells transfected with the let-7e-5p mimicking substrate was significantly lower than that in the negative control (p = 0.032) (**Figure 2F**). The fusion index of the let-7e-5p mimicking substrate was significantly less than that of the negative control (p = 0.041) or the inhibitor (p = 0.001) (**Figure 2G**). Additionally, myotube width of the C2C12 cells transfected with the let-7e-5p inhibitor was significantly higher than that with the negative control (p = 0.001) (**Figure 2H**). Moreover, the viability was investigated with Annexin V by flowcytometry. Then, the ratio of apoptosis and dead cells was increased by transfection with the let-7e-5p mimicking substrate, compared to negative control, whereas transfection with the let-7e-5p inhibitor significantly decreased the apoptosis and dead cells (p = 0.001) (**Figures 2I–L**). In addition, the fluorescence intensity of myogenin in the C2C12 cells transfected with the let-7e-5p inhibitor was significantly higher than that in the cells transfected with the negative control (p = 0.001), whereas the level in cells transfected with the let-7e-5p mimicking substrate was significantly lower than that in the negative control (p = 0.034) (**Supplementary Figures 4A, B**).

### Let-7e-5p Downregulated Glucose Uptake in C2C12 Cells

**Figure 3:**
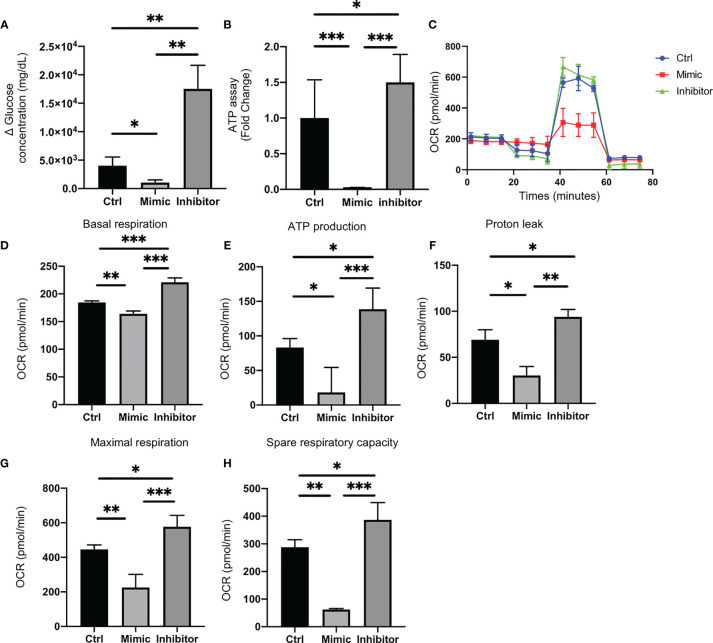
Let-7e-5p decreases glucose uptake and downregulates mitochondrial function in C2C12 cells. **(A)** Glucose uptake by C2C12 cells was monitored after transfection with let-7e-5p mimic/inhibitor. Glucose uptake was downregulated by let-7e-5p mimicking substrate and upregulated by let-7e-5p inhibitor, compared to negative control (n = 6). **(B)** ATP assay following transfection with let-7e-5p mimic/inhibitor. ATP production was downregulated by let-7e-5p mimicking substrate and upregulated by let-7e-5p inhibitor, compared to negative control (n = 6). **(C)** Raw data of oxygen consumption rate (OCR), **(D)** basal respiration, **(E)** ATP-linked respiration (oligomycin-sensitive OCR), **(F)** proton leak, **(G)** maximal mitochondrial respiration (FCCP-stimulated OCR), and **(H)** spare respiratory capacity. OCRs were normalized to the total number of cells. Data represent the means ± SD; *p = 0.05, **p = 0.01, ***p = 0.001 by one-way ANOVA.

Glucose uptake in C2C12 cells transfected with let-7e-5p inhibitor was significantly increased (p = 0.002), whereas that in cells transfected with let-7e-5p mimicking substrate was significantly decreased (p = 0.026) compared to the negative control (**Figure 3A**).

### Let-7e-5p Downregulated ATP Activity in C2C12 Cells

ATP activity in C2C12 cells transfected with let-7e-5p mimicking substrate was significantly downregulated, compared to that of the negative control (p = 0.001), whereas the ATP activity in cells transfected with let-7e-5p inhibitor was upregulated (p = 0.039) (**Figure 3B**). Next, we investigated the mitochondrial OCR of C2C12 cells using an extracellular flux analyzer. C2C12 cells transfected with let-7e-5p mimicking substrate showed decreased basal respiration. In addition, C2C12 cells transfected with let-7e-5p mimicking substrate had significantly decreased ATP production, proton leak, maximal respiration, and spare respiratory capacity, compared to negative control and cells transfected with let-7e-5p inhibitor. On the contrary, their mitochondrial function was upregulated by the inhibition of let-7e-5p (**Figures 3C–H**).

### Expression of Let-7e-5p in Serum of Patients With Muscle Atrophy Was Significantly Lower Than That in Patients Without Muscle Atrophy

**Figure 4:**
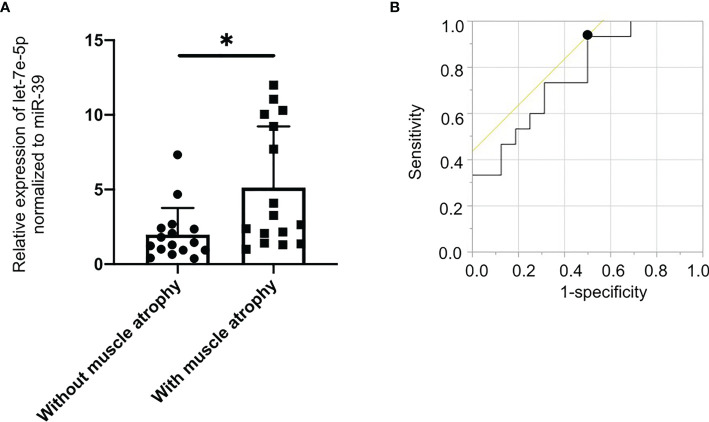
Decreased levels of serum circulating let-7e-5p in patients with muscle atrophy compared to patients without muscle atrophy. **(A) **Relative expression of serum circulating let-7e-5p normalized to miR-39 in the patients with and without muscle atrophy is shown. Serum let-7e-5p expression in patients without muscle atrophy (7.1 ± 10.1) was significantly higher than that in patients with muscle atrophy (2.0 ± 1.8) (p = 0.028). Data represent the means ± SD. **(B)** Optimal cut-off point for serum circulating let-7e-5p level, for the presence of sarcopenia, is 1.302 (AUC 0.767, 95% CI, 0.600–0.934, sensitivity = 0.933, specificity = 0.500, p = 0.001). *p = 0.05 by Pearson’s chi-square test.

Since let-7e-5p was suggested to have an effect on skeletal muscle in animal and cell experiments, we investigated serum let-7e-5p levels to test whether it is a biomarker for sarcopenia in our human studies. The characteristics of the 32 male patients with diabetes (with and without muscle atrophy) are summarized in **Table 1**. We investigated the differences in the expression of let-7e-5p in the serum between the two groups. Serum let-7e-5p expression in patients with muscle atrophy was significantly lower than that in patients without muscle atrophy (p = 0.028) (**Figure 4A**). Serum let-7e-5p level of 1.302 was identified as the cut-off for the presence of sarcopenia in the patients with diabetes (**Figure 4B**). Serum let-7e-5p level was found to be negatively associated with the presence of muscle atrophy in both univariate logistic regression analysis (OR of 1-unit increment: 0.70, 95% confidence interval (CI): 0.50–0.99, p = 0.011) and multivariate logistic regression analysis after adjusting for covariates (OR of 1-unit increment: 0.68, 95% CI: 048–0.99, p = 0.009) (**Table 2**).

**Table 1:** Characteristics of the study patients with and without muscle atrophy.

	With Muscle atrophy n=16	Without Muscle atrophy n=16	*p*-value
Age, yrs	69.8 (6.9)	69.4 (6.3)	0.817
SMI, kg/m^2^	5.7 (0.8)	7.8 (1.0)	< 0.001
Let-7e-5p	2.0 (1.8)	7.1 (10.1)	0.028

Data are mean (SD). SMI: skeletal muscle mass index.

**Table 2:** Logistic regression analyses for muscle atrophy.

	Univariate		Multivariate	
	OR (95%CI)	*p*-value	OR (95%CI)	*p*-value
Age, yrs	1.01 (0.91-1.11)	0.881	1.04 (0.92-1.17)	0.507
Let-7e-5p	0.70 (0.50-0.99)	0.011	0.68 (0.48-0.99)	0.009

Multivariate analysis was adjusted for age.

CI, confidential interval; OR, odds ratio.

## Discussion

Androgen deficiency is known to be associated not only with muscle atrophy (31), but also with insulin resistance, type 2 diabetes, metabolic syndrome, and visceral fat accumulation (32, 33). In the present study, the expression of the miRNA, let-7e-5p, was found to be decreased in the soleus muscle of ORX mice compared to that in Sham mice, whereas replacement of testosterone restored the expression of let-7e-5p. Furthermore, let-7e-5p was found to promote muscle atrophy by inhibiting the function of Igf2bp2, thereby reducing glucose uptake by myotube cells, and thus impairing mitochondrial function. In addition, serum let-7e-5p levels were significantly lower in patients with muscle atrophy than in those without. In this study, we also identified that the cut-off value of serum let-7e-5p level in patients with diabetes, for the presence of muscle atrophy, was 1.302.

The lethal-7 (let-7) gene was first discovered as an important developmental regulator in Caenorhabditis elegans. Let-7e-5p inhibits proliferation and metastasis of glioma stem cells (34) and colorectal cancer (35), and its functions are being intensively investigated in the field of oncology. Several target genes of let-7e-5p have been identified. In this study, we focused on Igf2bp2 and found that let-7e-5p regulates Igf2bp2. Igf2bps 1, 2, and 3 belong to a highly conserved family of RNA-binding proteins that influence the fate of transcripts (36–38). In contrast to Igf2bp1 and Igf2bp3, which are expressed during development, Igf2bp2 is widely expressed in many adult tissues, including the gut, muscles, and brain. In these organs, small quantitative differences in Igf2bp2 expression subtly affects processes such as food uptake, metabolism, feeding behavior, or more complex behavioral features that affects physical activity and, ultimately, the lifetime risk of developing obesity and diabetes (39). In their study of the relationship between IGFBP2 and skeletal muscle, Caron et al. (40) demonstrated that overexpression of high mobility group A2, an upstream target of Igf2bp2, enhanced myogenesis and myosin heavy chain expression in embryonic stem cells. Furthermore, since the muscle atrophy-related genes investigated in this study, such as A, B, and C, do not share a common binding site with let-7e-5p, we hypothesized that the gene expression of IGF2BP2 indirectly alters the expression of these muscle atrophy-related genes. Moreover, Igf2bp2 promotes translation of IGF2 through internal ribosomal entry and downstream PI3K/Akt signaling (41), which inhibits FOXO1 and MURF1, and suppresses muscle atrophy (42). A previous study has shown that Igf2bp2 functions as a key regulator of satellite cell activation and skeletal muscle development (43). Adjusting the expression of let-7e-5p altered the expression of myogenin, one of the differentiation markers of myotubular cells (44), suggesting that let-7e-5p may also be involved in muscle differentiation through the regulation of IGF2BP2 expression. In addition, the other previous study reported that postnatal Igf2bp2 inactivation in mouse skeletal muscles reduces muscle mass accompanied by a reduction in overall protein synthesis due to reduced autocrine production of IGF2 and impaired activation of Akt1, Gsk3α, and eIF2Bϵ (45). In this study, suppression of let-7e-5p in C2C12 cells significantly increased the expression of Igf2bp2, which resulted in muscle hypertrophy. Furthermore, Igf2bp2 promotes the transport of mRNAs into the vicinity of mitochondria, and subsequent translation and mediation of cellular functions (46). In contrast, the suppression of Igf2bp2 reduces the OCR (47). In fact, in our study, mitochondrial function was enhanced by the suppression of let-7e-5p in C2C12 cells.

Serum let-7e-5p levels were significantly lower in diabetic patients with sarcopenia compared to that in patients without sarcopenia. This suggests that serum let-7e-5p level can be used as a diagnostic marker for sarcopenia. We hypothesize that serum let-7e-5p levels in patients were lower to reduce atrophy of skeletal muscles caused by deficiency in androgen. However, it is difficult to derive a causal relationship from this cross-sectional analysis. Therefore, we intend to perform longitudinal analyses to understand this relationship further in future studies.

Our study has the following limitation. The effects of let-7e-5p on skeletal muscles was only demonstrated through cellular experiments in this study. In the future, we intend to examine the influence of impaired glucose tolerance on skeletal muscles in animal experiments using conditional knockout mice. In addition, the possibility of mRNA-miRNA interaction needed to be evaluated not only by dual-luciferase assay but also by ribonucleoprotein immunoprecipitation.

In conclusion, this study revealed that the overexpression of Igf2bp2 in C2C12 cells using a let-7e-5p inhibitor improves sarcopenia mainly *via* suppression of genes associated with muscle atrophy and enhanced mitochondrial function. Therefore, inhibition of let-7e-5p in skeletal muscles represents a potential therapeutic target for sarcopenia. Additionally, serum let-7e-5p level may be used as a marker for sarcopenia.

## Data Availability Statement

The data that support the findings of this study are available from the corresponding author upon reasonable request.

## Ethics Statement

The studies involving human participants were reviewed and approved by the research ethics committees of the Kyoto Prefectural University of Medicine and Kameoka Municipal Hospital. The patients/participants provided their written informed consent to participate in this study. The animal study was reviewed and approved by the Committee for Animal Research, Kyoto Prefectural University of Medicine.

## Author Contributions

TO originated and designed the study, researched the data, and wrote the manuscript. HO and YH originated and designed the study, researched the data, and reviewed the manuscript. HO, YH, and MH researched the data and contributed to the discussion. MF originated and designed the study, researched the data, and reviewed and edited the manuscript. MF is the guarantor of this work and, as such, had full access to all of the data in the study and takes responsibility for the integrity of the data and the accuracy of the data analysis. All authors were involved in the writing of the manuscript and approved the final version of this article.

## Funding

This work was supported by KAKENHI, Grant-in-Aid for Young Scientists (19K17966).

## Conflict of Interest

YH has received grants from Asahi Kasei Pharma, personal fees from Daiichi Sankyo Co., Ltd., personal fees from Mitsubishi Tanabe Pharma Corp., personal fees from Sanofi K.K., personal fees from Novo Nordisk Pharma Ltd., outside the submitted work. TS has received personal fees from Ono Pharma Co., Ltd., Mitsubishi Tanabe Pharma Co, Astellas Pharma Inc., Kyowa Hakko Kirin Co., Ltd., Sanofi K.K., MSD K.K., Kowa Pharma Co., Ltd., Taisho Toyama Pharma Co., Ltd., Takeda Pharma Co., Ltd., Kissei Pharma Co., Ltd., Novo Nordisk Pharma Ltd., Eli Lilly Japan K.K. outside the submitted work. EU has received grants from the Japanese Study Group for Physiology and Management of Blood Pressure, the Astellas Foundation for Research on Metabolic Disorders (Grant number: 4024). Donated Fund Laboratory of Diabetes therapeutics is an endowment department, supported with an unrestricted grant from Ono Pharmaceutical Co., Ltd., and received personal fees from AstraZeneca plc, Astellas Pharma Inc., Daiichi Sankyo Co., Ltd., Kyowa Hakko Kirin Company Ltd., Kowa Pharmaceutical Co., Ltd., MSD K.K., Mitsubishi Tanabe Pharma Corp., Novo Nordisk Pharma Ltd., Taisho Toyama Pharmaceutical Co., Ltd., Takeda Pharmaceutical Co., Ltd., Nippon Boehringer Ingelheim Co., Ltd., and Sumitomo Dainippon Pharma Co., Ltd., outside the submitted work. MH has received grants from Asahi Kasei Pharma, Nippon Boehringer Ingelheim Co., Ltd., Mitsubishi Tanabe Pharma Corporation, Daiichi Sankyo Co., Ltd., Sanofi K.K., Takeda Pharmaceutical Company Limited, Astellas Pharma Inc., Kyowa Kirin Co., Ltd., Sumitomo Dainippon Pharma Co., Ltd., Novo Nordisk Pharma Ltd., and Eli Lilly Japan K.K., outside the submitted work. MA received personal fees from Novo Nordisk Pharma Ltd., Abbott Japan Co., Ltd., AstraZeneca plc, Kowa Pharmaceutical Co., Ltd., Ono Pharmaceutical Co., Ltd., Takeda Pharmaceutical Co., Ltd., outside the submitted work. MY reports personal fees from MSD K.K., Sumitomo Dainippon Pharma Co., Ltd., Kowa Company, Limited, AstraZeneca PLC, Takeda Pharmaceutical Company Limited, Kyowa Hakko Kirin Co., Ltd., Daiichi Sankyo Co., Ltd., Kowa Pharmaceutical Co., Ltd., Ono Pharma Co., Ltd., outside the submitted work. MF has received grants from Nippon Boehringer Ingelheim Co., Ltd., Kissei Pharma Co., Ltd., Mitsubishi Tanabe Pharma Co, Daiichi Sankyo Co., Ltd., Sanofi K.K., Takeda Pharma Co., Ltd., Astellas Pharma Inc., MSD K.K., Kyowa Hakko Kirin Co., Ltd., Sumitomo Dainippon Pharma Co., Ltd., Kowa Pharmaceutical Co., Ltd., Novo Nordisk Pharma Ltd., Ono Pharma Co., Ltd., Sanwa Kagaku Kenkyusho Co., Ltd. Eli Lilly Japan K.K., Taisho Pharma Co., Ltd., Terumo Co., Teijin Pharma Ltd., Nippon Chemiphar Co., Ltd., and Johnson & Johnson K.K. Medical Co., Abbott Japan Co., Ltd., and received personal fees from Nippon Boehringer Ingelheim Co., Ltd., Kissei Pharma Co., Ltd., Mitsubishi Tanabe Pharma Corp., Daiichi Sankyo Co., Ltd., Sanofi K.K., Takeda Pharma Co., Ltd., Astellas Pharma Inc., MSD K.K., Kyowa Kirin Co., Ltd., Sumitomo Dainippon Pharma Co., Ltd., Kowa Pharma Co., Ltd., Novo Nordisk Pharma Ltd., Ono Pharma Co., Ltd., Sanwa Kagaku Kenkyusho Co., Ltd., Eli Lilly Japan K.K., Taisho Pharma Co., Ltd., Bayer Yakuhin, Ltd., AstraZeneca K.K., Mochida Pharma Co., Ltd., Abbott Japan Co., Ltd., Medtronic Japan Co., Ltd., Arkley Inc., Teijin Pharma Ltd. and Nipro Cor., outside the submitted work.

The remaining authors declare that the research was conducted in the absence of any commercial or financial relationships that could be construed as a potential conflict of interest.

## Publisher’s Note

All claims expressed in this article are solely those of the authors and do not necessarily represent those of their affiliated organizations, or those of the publisher, the editors and the reviewers. Any product that may be evaluated in this article, or claim that may be made by its manufacturer, is not guaranteed or endorsed by the publisher.
