# Supplementary figures and images for: Let-7e-5p Regulates IGF2BP2, and Induces Muscle Atrophy
Source: Front Endocrinol (Lausanne). 2021 Dec 24;12:791363. doi: 10.3389/fendo.2021.791363 (PMC8741024; doi:10.3389/fendo.2021.791363)

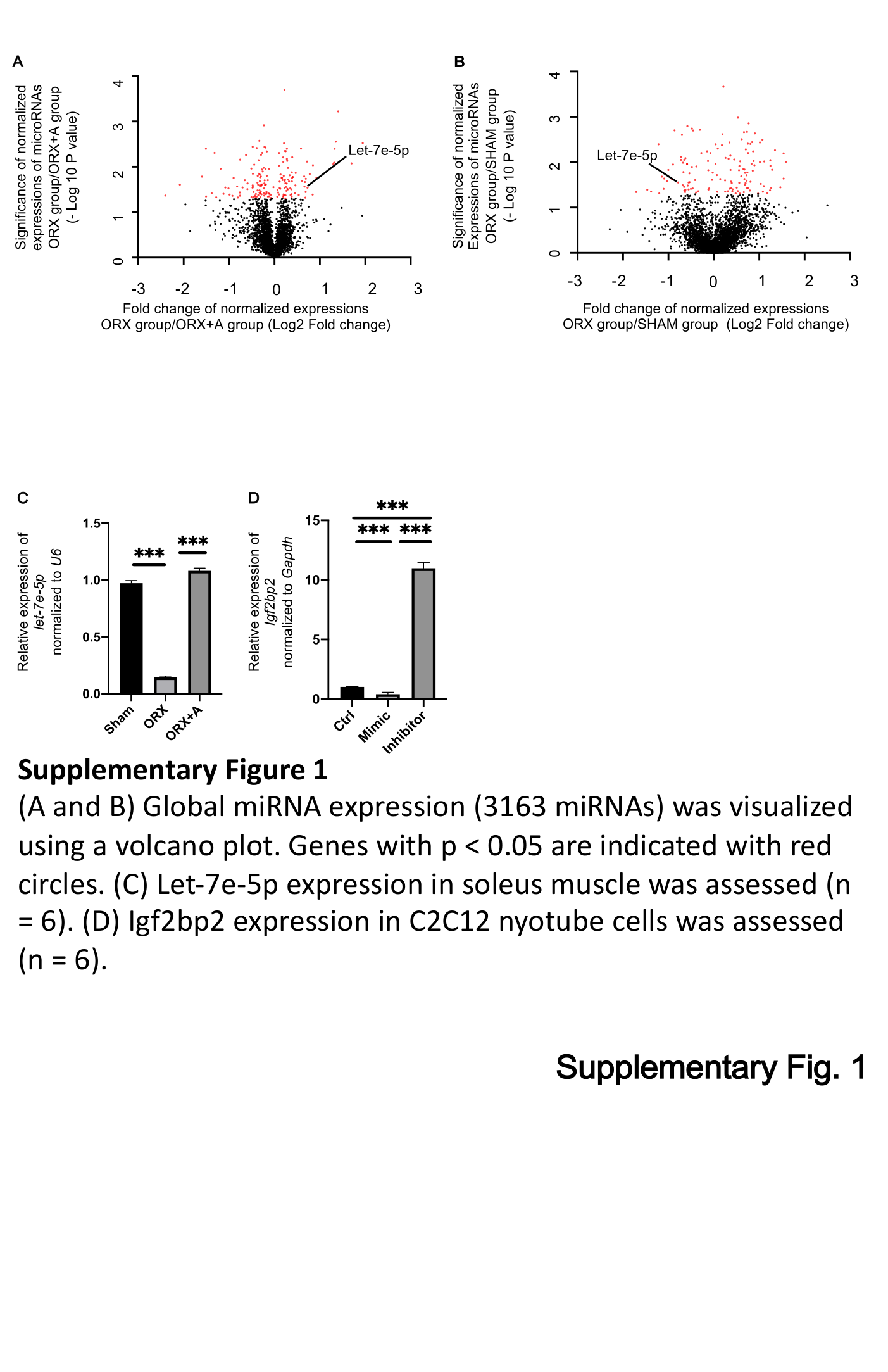

Supplement: Supplementary file 1 [file Image_1.tiff]

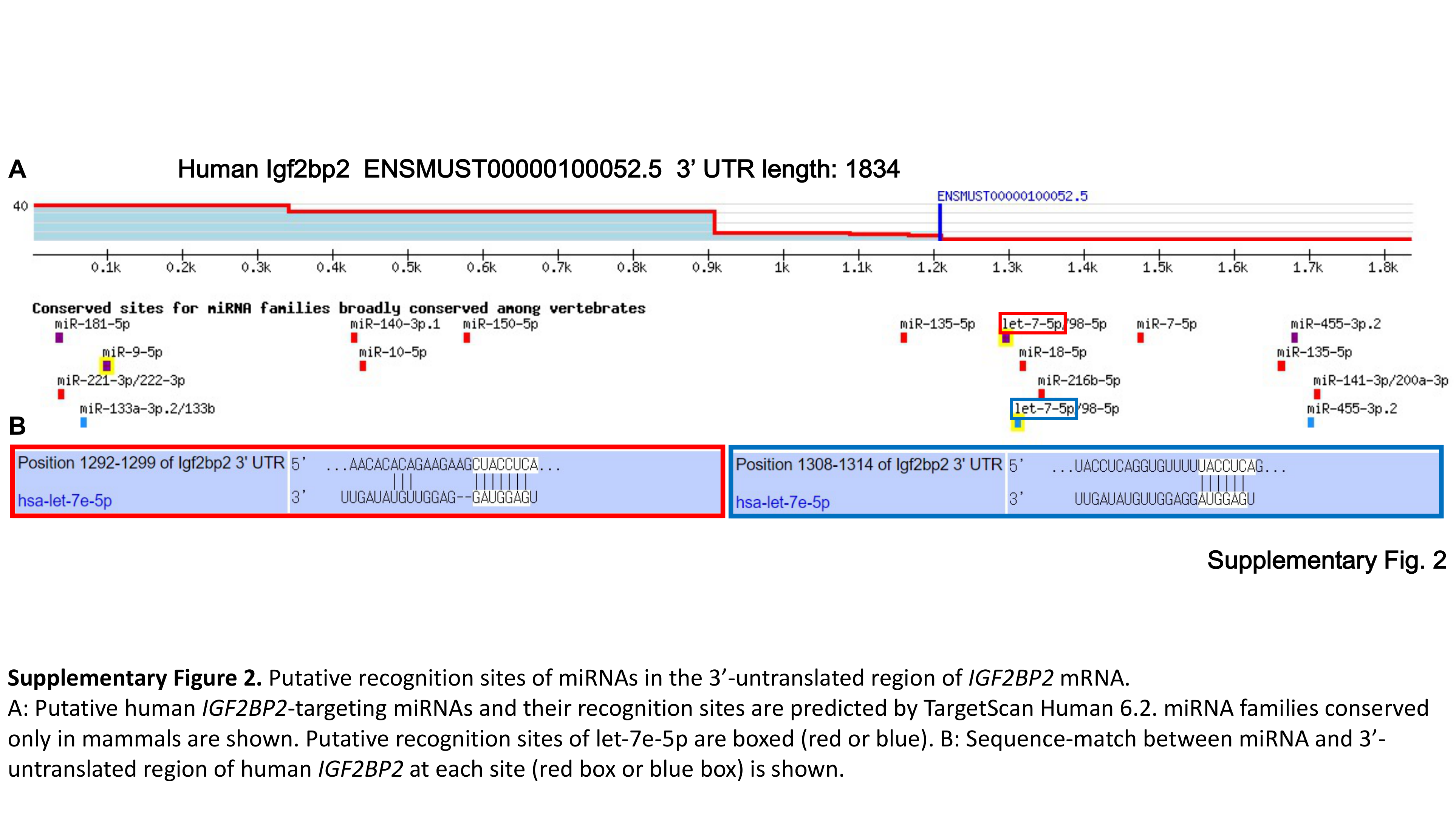

Supplement: Supplementary file 2 [file Image_2.tiff]

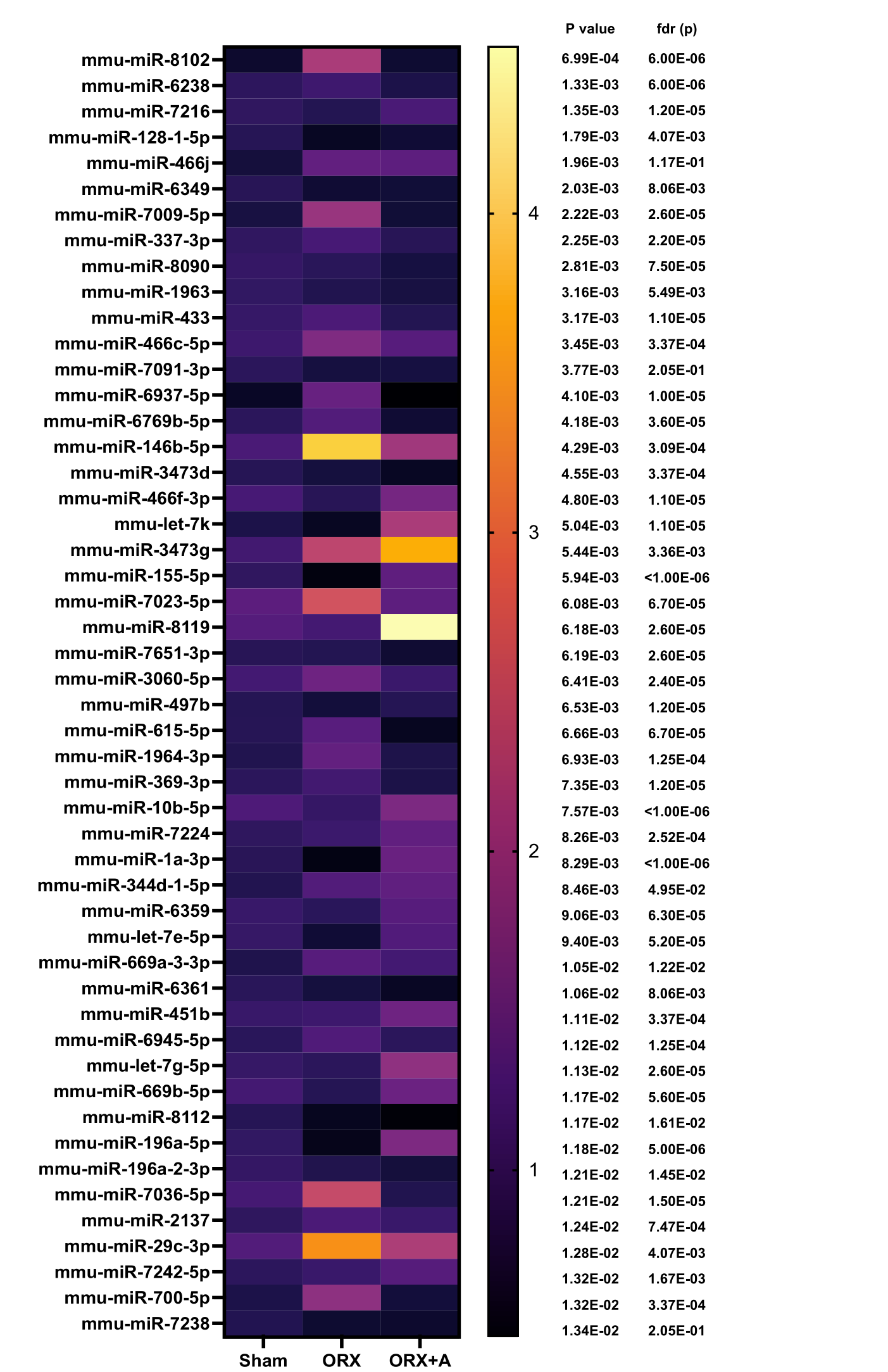

Supplement: Supplementary file 3 [file Image_3.tiff]

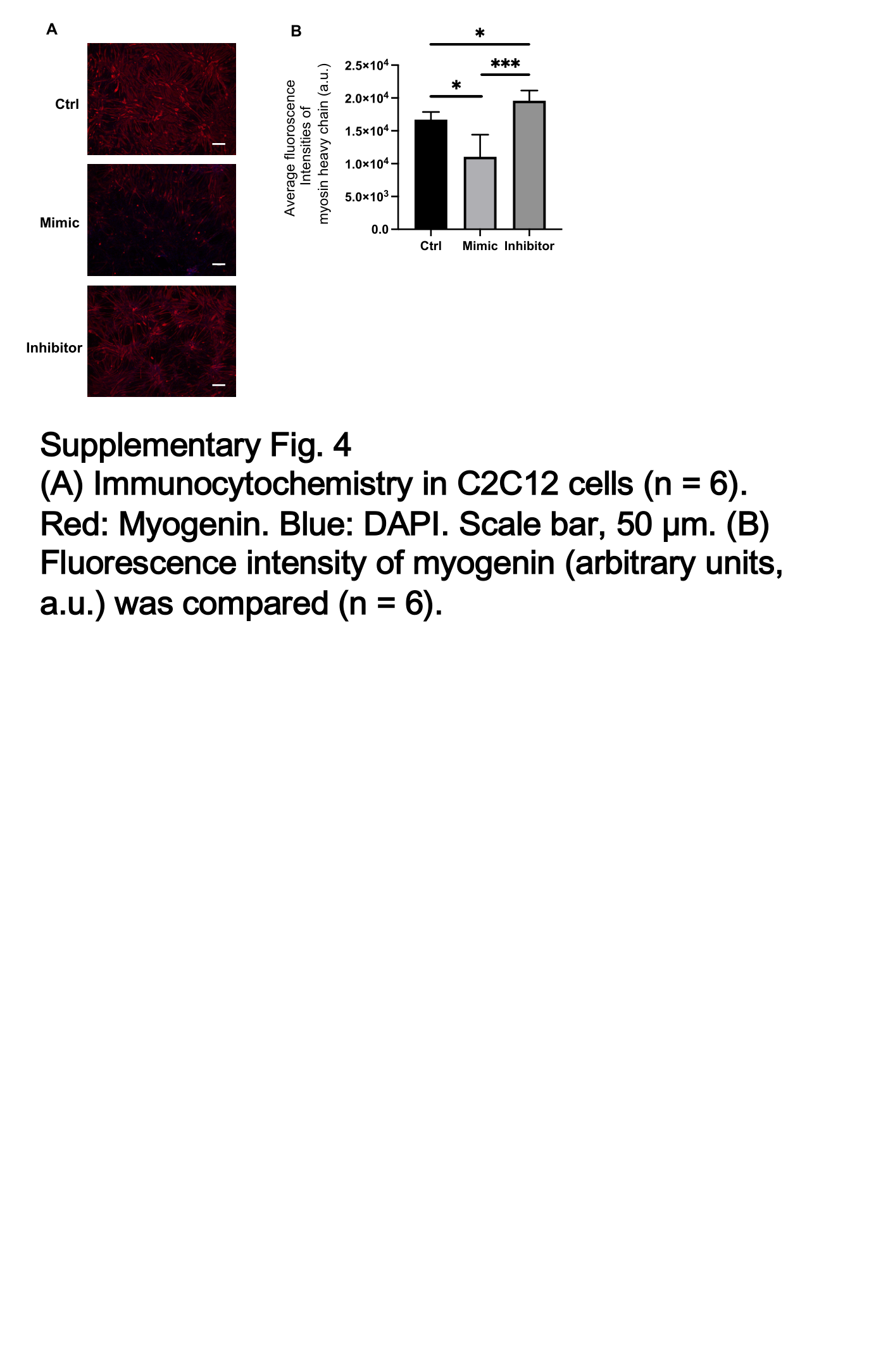

Supplement: Supplementary file 4 [file Image_4.tiff]
